# Incorporation of local structure into kriging models for the prediction of atomistic properties in the water decamer

**DOI:** 10.1002/jcc.24465

**Published:** 2016-08-18

**Authors:** Stuart J Davie, Nicodemo Di Pasquale, Paul L. A. Popelier

**Affiliations:** ^1^ Manchester Institute of Biotechnology (MIB), 131 Princess Street, Manchester M1 7DN, Great Britain and School of Chemistry, University of Manchester, Oxford RoadManchesterM13 9PLGreat Britain

**Keywords:** force field design, liquid water, interacting quantum atoms, quantum chemical topology, quantum theory of atoms in molecules, machine learning, kriging

## Abstract

Machine learning algorithms have been demonstrated to predict atomistic properties approaching the accuracy of quantum chemical calculations at significantly less computational cost. Difficulties arise, however, when attempting to apply these techniques to large systems, or systems possessing excessive conformational freedom. In this article, the machine learning method kriging is applied to predict both the intra‐atomic and interatomic energies, as well as the electrostatic multipole moments, of the atoms of a water molecule at the center of a 10 water molecule (decamer) cluster. Unlike previous work, where the properties of small water clusters were predicted using a molecular local frame, and where training set inputs (*features*) were based on atomic index, a variety of feature definitions and coordinate frames are considered here to increase prediction accuracy. It is shown that, for a water molecule at the center of a decamer, no single method of defining features or coordinate schemes is optimal for every property. However, explicitly accounting for the structure of the first solvation shell in the definition of the features of the kriging training set, and centring the coordinate frame on the atom‐of‐interest will, in general, return better predictions than models that apply the standard methods of feature definition, or a molecular coordinate frame. © 2016 The Authors. Journal of Computational Chemistry Published by Wiley Periodicals, Inc.

## Introduction

Molecular dynamics (MD) simulations are an important tool in understanding the dynamical evolution of condensed matter systems. However, as many important properties of condensed matter occur over long time scales, the development of both accurate and efficient means to calculate atomic interactions is essential. In contrast to computationally expensive *ab initio* MD techniques,^1^, ^2^ most condensed phase simulations currently rely on one of a number of parameterizable force fields, including CHARMM^3^ and AMBER,^4^ among others. Such force fields commonly treat electrostatics as pairwise interactions between point charges, and may describe the energy variation of a molecular bond under compression or elongation through a simple Hooke potential. While many‐body effects, and/or polarization, can be effectively included, such potentials are still some way off being able to reproduce the bulk properties of many systems (including liquid water^5^, ^6^) and suffer an extensive parameterization challenge in reactions that involve the breaking or formation of covalent bonds.

Difficulties in force field parameterization are particularly evident in the case of water. Water, despite being the subject of extensive research due to its fundamental role in life on earth, has peculiar characteristics not yet fully understood. The structure of water was included among the 125 most important questions in modern science according to Science.^7^ Lack of replication of water's structure and properties in simulation has led to the development of multiple water specific force fields over the last 30 years.^8^ Most commonly, force fields for water are normally optimized against water's bulk properties, by fine‐tuning parameters used to define pairwise interactions. Unfortunately, such simple models usually lack polarization, and nonpolarizable force fields designed for water through parameter optimization are only able to accurately predict a subset of water's properties.^9^ Furthermore, the necessity of including the underlying quantum mechanical effects in the description of simulated water molecules has been acknowledged by several authors.^10^, ^11^

An alternative approach to force field design, known as the quantum chemical topology force field (QCTFF),^12^ sees individual atoms as malleable boxes of electron density as defined by the quantum theory of atoms in molecules (QTAIM),^13^, ^14^ which interact at long range through multipolar electrostatics, and at short range through interatomic Coulomb, exchange and correlation energies as defined through the interacting quantum atoms (IQA) energy partitioning scheme.^15^ The properties of these malleable boxes, including the IQA energies, are efficiently predicted on the fly through the use of the machine learning method kriging.^16^, ^17^, ^18^ At its limit, kriging returns near quantum mechanical accuracy, and includes polarization (as an end effect rather than the polarizability^19^ itself) and many‐body effects. There is no need for additional *ab initio* calculations once each kriging model is trained. In our work, kriging achieves this by successfully mapping the outputs (atomic energies and multipole moments) *directly* to the inputs, also called *features* (geometrical parameters based on the nuclear coordinates of the surrounding atoms).

Although the application of machine learning to computational chemical calculations is rapidly gaining popularity, there are still many problems yet to be resolved. For example, until now, many machine learning models have either focused on predicting the properties of a collection of small molecules *in vacuo* 20, 21; the properties of a larger molecule undergoing some form of perturbation (for example, through the distortion of the molecule's normal modes of vibration)^22^, ^23^, ^24^; or a combination of both.^25^, ^26^ Unfortunately, such approaches do not adequately sample the extensive conformational freedom of a cluster of MD sampled water molecules—a challenge that must be met if the QCTFF is to be able to simulate solvated systems. Previous work by Handley et al.^27^, ^28^ has applied kriging models to predict the electrostatic energies of MD sampled water clusters up to the hexamer, while Mills et al. applied kriging to predict the electrostatic energy of a hydrated sodium ion.^29^ Even within the regime of small clusters (≤ 6 water molecules) difficulties were experienced, with Hawe and Popelier^30^ recommending seven distinct models be used to cover conformational space of an MD sampled water dimer, in order to reduce maximum electrostatic errors.

It is important that the properties of larger water clusters begin to be addressed, for the future goal of using machine learning to predict bulk fluid. Consideration of clusters larger than the first solvation shell can be justified by two factors: (i) the long range cooperative effects of water's hydrogen bonding networks,^31^ and (ii) the non‐negligible influence that the addition of water molecules, beyond the first solvation shell, has on a central molecule's charge distribution.^32^ In this work, kriging is applied to predict the IQA energies, and the QTAIM obtained multipole moments, of a central water molecule in an MD sampled decamer. Unlike previous QCTFF work, the MD sampled decamers possess excessive conformational freedom, rendering standard methods of training set (i.e., the list of features used as the model input) construction ineffective. Accordingly, 12 different training sets per property were constructed using different feature‐defining methods or different coordinate frames, in an attempt to accommodate the local structure of the cluster's hydrogen bonding network, and were contrasted with the prediction statistics obtained from applying the standard method. The effect of applying multiple, individual, atom‐centered coordinate frames, as opposed to using a single coordinate frame for the properties of both the oxygen and hydrogen atoms (in effect, a molecule centered coordinate frame, as per Refs. [ 25, 26, 28]), was also investigated.

This article is organized as follows. After a brief introduction of the QTAIM and IQA model, we describe the machine learning technique we are using, called kriging. We then describe how the decamer system is sampled and how predictions are obtained. Results from the application of the kriging method to the prediction of the multipole moments and IQA energies are then shown. Finally, major conclusions of the present work are summarized in the last section.

## Method

### QTAIM and IQA

The QTAIM is an atomic partitioning scheme, that divides a molecule into a collection of space‐filling, nonoverlapping topological atoms based on the gradient of the system's electron density.^13^, ^14^ With each atom assigned an atomic basin of electron density, various atomic and interatomic properties can be derived. For example, the electron density of each atomic basin can be accurately modeled using atom‐centered multipole moments.

Using the atomic basins of the partitioned molecule, IQA partitions the total wave function energy of the system into a sum of atomic self‐energies, $E_{Self}$, (which is also called the intra‐atomic energy) and interatomic interaction energies, $E_{Inter}$,

(1) ETotal=∑AESelfA+∑A>BEInterABEE=∑ATA+VneAA+VeeAA+∑A>BVnnAB+VneAB+VneBA+VeeAB

where $T^{A}$ is the kinetic energy of atom *A*; $V_{ne}^{AA}$ is the energy due to nuclear–electron interactions within the atomic basin of *A;* $V_{ne}^{AB}$ is the nuclear–electron interaction energy between the nucleus *A* and the electrons in the basin of *B*, and $\;V_{ne}^{BA}$ the opposite; $V_{ee}^{AA}$ and $V_{ee}^{AB}$ are the energy due to electron‐electron interactions within the atomic basin of *A*, and between basins *A* and *B,* respectively; and $V_{nn}^{AB\;}$ is the energy due to nuclear–nuclear interaction between *A* and *B*. As the electron density of a molecular system is a function of the system's atomic configuration,^33^ such multipole moments are well suited to machine learning techniques.

### Kriging

Kriging is a machine learning technique capable of interpolation, mapping an output's response to a given set of inputs. Predictions at an unknown position can then be obtained by using correlations among the property‐of‐interest in known locations. Intuitively, kriging assumes the smoothness of physical phenomena in space by considering that the value of a property in a given location is more likely to be close in value to the neighborhood's points, rather than far away ones. The predicted output, $\hat{y}$, for a given set of inputs $x^{*}$, is given through the equation^16^, ^17^, ^18^:

(2) $$\hat{y}(x^{*})=\hat{\mu }+\sum_{i=1}^{n}a_{i}\cdot \phi (x^{*}-x^{i})$$

where $\hat{\mu }$ models the global mean of the training data, a is a vector of constant weights, and $\phi \left(x^{*}-x^{i}\right)$ is a basis function relating the input $x^{*}$ to the i=1⇒n training point $x^{i}$. In this work, we use the following basis function:

(3) $$\phi (x^{*}-x^{i})=exp[-\sum_{h=1}^{d}\theta_{h}{\left|x_{h}^{*}-x_{h}^{i}\right|}^{p_{h}}]$$

where *d* is the number of features describing the system (i.e., the dimensionality of the problem), and where $\theta_{h}$ and $p_{h}$ are hyper‐parameters corresponding to feature *h*, and are obtained through maximizing the log‐likelihood function:

(4) $$L\left(\theta ,p,\sigma ,\mu \right)=-\frac{N_{t}}{2}ln(\sigma^{2})-\frac{1}{2}ln(|R|)-\frac{{\left(y-1\mu \right)}^{T}R^{-1}(y-1\mu )}{2\sigma^{2}}$$

where ***y*** is the column vector of the modeled property evaluated at each of the *N_t_* training points; 1 is a column vector of ones; ${\left(\cdot \right)}^{T}$ is the transpose of its argument, $\sigma^{2}$ is the variance; μ is the mean; R is the correlation matrix, where element $ij=\phi (x^{i}-x^{j})$, and $\left|R\right|$ its determinant.

Conceptually, eq. (2) can be thought of as predicting property *y* from a combination of the global mean of the property, plus an error term that is correlated to the surrounding points used to train the model. As the point to be predicted approaches a training point (i.e., $x^{*}\Rightarrow x^{i}$), it can be seen from eq. (3) that the correlation between the test point and training point will increase to one. This gives simple kriging the attractive property of being able to perfectly predict any test point that has the exact same input coordinates as one of the model's training points. Obtaining kriging weights requires the optimization of eq. (4), a formidable problem that scales quickly with the dimension of the system and the number of training points considered. In fact, calculation of the log‐likelihood requires the computation, and inversion, of the ***R*** matrix, which can render iterative algorithms computationally infeasible. Recent work^34^ has demonstrated the reliability of both particle swarm optimization and differential evolution regards maximizing eq. (4) for systems as large as a water hexamer. The optimization parameters recommended in Ref. [ 32] were used in this work, but $p_{h}$ was fixed to 2 (making eq. (3) the “Gaussian basis function”) for all *h* to increase efficiency.

### Sampling

A set of 5000 water clusters, composed of ten molecules (from now on known as 'decamers'), was sampled by selecting the nine nearest neighbors of a water molecule at intermittent snapshots from a previous MD simulation completed at room temperature and with multipole moments.^35^ The simulations were completed using the MD package DL_POLY_2.0,^36^ with the water molecules constrained to approximate rigid‐bodies. Slight fluctuations in the intramolecular bond lengths still occurred, due to a relatively low quaternion tolerance. These fluctuations, as well as the statistical properties of the distance from the central water molecule to the furthest water molecule are displayed in Table 1. The wave function for each cluster was calculated using the GAUSSIAN09^37^ package at the B3LYP/6‐311++G(d,p) level of theory. Although the water molecules were constrained to approximate rigid bodies in the MD simulation, the slight fluctuations in bond lengths and angles were enough to result in a range of 0.5 kJ/mol in wave function energies for the water molecules if considered as isolated monomers. The program AIMAll^38^ was then used to obtain the atomic multipole moments, as well as the IQA energies,^39^ using default settings and integration error control. In Ref. [ 39] IQA energy contributions were explicitly reconciled with the B3LYP functional, thereby recovering for the first time the total energy when using B3LYP. This approach chose to use the explicit B3LYP functional only within a single atom, that is, for the total atomic energy only. Conversely, the Hartree–Fock‐like expression was adopted for interatomic exchange energy but then using Kohn–Sham orbitals. In other words, this approach calculates the interatomic exchange–correlation contribution ( $V_{\text{XC}}^{\text{AB}}$) via the pure Hartree–Fock exchange equation only, but by inserting KS orbitals instead of HF orbitals (see eq. (14) in Ref. [ 39]).

**Table 1:** Configuration statistics of set of decamer clusters.

	Intramolecular O—H distance (Å)	Intramolecular HOH angle (degrees)	Largest O—O distance (Å)
**Mean**	0.9583	104.45	4.096
**Std Dev**.	0.0005	0.06	0.260
**Range**	0.011	0.86	1.539

### Standard kriging models

The atomic configuration of each cluster was used to provide the features for the kriging models. In previous QCTFF work, a variety of coordinate frames have been used to describe the selected systems of interest.^23^, ^28^, ^29^ Although similar systems have defined coordinate frames that implicitly account for the rigidity of the surrounding water molecules^28^, ^29^ an atomistic coordinate frame was used here as it both accounts for the energetic fluctuations caused by the minor intramolecular distortions present, and being general, allows readily for application to fully flexible water molecules.

**Figure 1:**
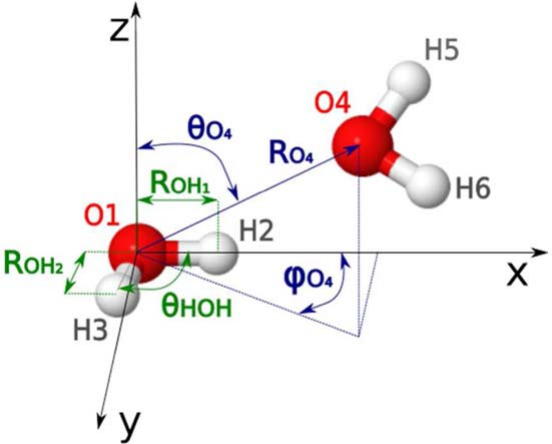
Coordinate frame and first six kriging features of a water molecule of interest and its neighbor. The *x*‐axis of the system is defined by the O_1_—H_2_ bond, and the *xy*‐plane is defined by the H_2_—O_1_—H_3_ angle. The first three features of the system correspond to $R_{{OH}_{1}},R_{{OH}_{2}},\theta_{HOH}$, respectively. The next atom by index (i.e., O_4_ in this example), is the next atom to be described in features, and is described by the three features $R_{O_{4}},\theta_{O_{4}},\varphi_{O_{4}}$. This convention is continued until every atom in the system is described. [Color figure can be viewed in the online issue, which is available at wileyonlinelibrary.com.]

Standard kriging models were created using a spherical polar coordinate frame centered on the oxygen of the central water molecule, with the *x*‐axis of the system defined along one of the molecule's OH bonds, and the *xy*‐plane defined to include the remaining hydrogen. A graphical depiction of the coordinate frame, and its corresponding features, is displayed in Figure 1. As per previous work using an atom‐centered, spherical polar coordinate frame,^23^, ^24^, ^26^ the first three features of the kriging training set, $R_{{OH}_{1}},R_{{OH}_{2}},\theta_{HOH}$, correspond to the two central OH bond lengths and the central water molecule's HOH angle. External atoms were each described by a set of three features, $R_{N},\theta_{N},\phi_{N}$ for atom *N*, where $R_{N}$ is the distance from the central oxygen to atom *N*, $\theta_{N}$ is the polar angle of atom *N* (measured from the *z*‐axis), while $\phi_{N\;}$ is the azimuthal angle of atom *N* (as measured from the *x‐*axis). Such a scheme results in a total of 3N−6 features. Thus, for the water dimer displayed in Figure 1, there will be 12 features: $\left\{R_{{OH}_{1}},R_{{OH}_{2}},\theta_{HOH},\;R_{O_{4}},\theta_{O_{4}},\phi_{O_{4}},\;R_{H_{5}},\theta_{H_{5}},\phi_{H_{5}},\;R_{H_{6}},\theta_{H_{6}},\phi_{H_{6}}\right\}$. According to the method used in previous work,^23^, ^24^, ^26^ the features describing the system were defined by order of atomic index (i.e., the order the atoms were listed in the simulation output). As per common practice, hydrogen atoms were indexed immediately after the oxygen atom they were bonded to, resulting in sets of nine features, adjacent in the training set, which completely describe the position of a single water molecule.

### *Standard* results

Kriging models were created using the *Standard* training set to predict the IQA energies of the atoms of the central water molecule, with Table 2 displaying the performance statistics of the results. Each kriging model was trained on the dataset of 5000 training points using fivefold cross validation, using a 1:4 partitioning of the training set to test set. In other words, kriging models were trained with 1000 randomly selected training points, with the remaining 4000 points used as a validation set; and that this process was repeated five times for statistical significance, while ensuring no training point was used in more than one model. The kriging models are compared through the mean absolute errors (MAE) obtained by comparing the predicted property values to the corresponding true property values, and through the *q* ^2^ correlation coefficient:

(5) $$q^{2}=1-\frac{\sum_{i=1}^{N_{test}}{\left(P_{i}-T_{i}\right)}^{2}}{\sum_{i=1}^{N_{test}}{\left(M-T_{i}\right)}^{2}}$$

where *N*_test_ is the number of points (i.e., 4000) the model is tested on; *P_i_* is the predicted value of test point *i*; *T_i_* is the true value of test point *i*; and *M* is the mean of the entire test set. Thus, the *q* ^2^ metric has the intuitive property of being equal to one when predictions are equal to true values ( $P_{i}-T_{i}=0$), and equal to zero when predictions are no better than the predictions obtained by using the simplest, unbiased estimator—the mean (i.e., when $P_{i}-T_{i}=M-T_{i}$). In other words, the 5000 training points were evenly split into five 1000‐training‐point models, with each model being tested on the set of points not used for that specific model. This resulted in five MAE and *q* ^2^ values per model, for which the average and standard deviation were calculated, and reported in Table 2.

**Table 2:** Prediction statistics for $E_{Self}$ and $E_{Inter}$ of the atoms of the central water molecule of a water decamer, created using the *Standard* training set.

Property	Mean Value	Range	MAE	*q* ^2^
Oxygen $E_{Self}$	−196708.8	243.9	35.2 ± 1.3	0.00 ± 0.00
Oxygen $E_{Inter}$	−1540.6	407.7	50.2 ± 2.1	0.00 ± 0.00
Hydrogen $E_{Self}$	−736.1	121.6	18.1 ± 0.2	0.00 ± 0.00
Hydrogen $E_{Inter}$	−525.3	120.8	18.4 ± 0.5	0.00 ± 0.00

All energies are in kJ/mol, and uncertainties express ± 1 standard deviation. Averages obtained from five 1000‐training‐point models, tested on 4000 test points each.

As seen in Table 2, the models created using the *Standard* training perform poorly. In fact, as each model returned a *q* ^2^ value of 0.0, the kriging models used here predict with no better accuracy than what would be predicted by the mean. This is because the model predicted very close to the mean for most test points; an expected result for systems trained with exceedingly few data points.

Note, however that the models do not exactly predict the mean for every test point. The results presented here are to two significant figures, and as such the fraction in eq. (5) is only equal to one to two significant figures. Thus, it can be concluded that using the standard method of training set construction for QCTFF kriging models does not produce useful kriging models for a MD sampled water decamer when sampled with 1000 training points.

### Distance‐defined kriging models

Increasing the number of training points in a kriging model is a trivial way to improve prediction accuracy.^17^, ^18^ Such a method works because an increased density of training data provides more information (in the form of higher correlations) to the kriging predictor, when attempting to predict the value of a property at a given point. In fact, as simple kriging is an interpolating predictor, the error on a prediction will approach zero as the distance between the prediction point of interest and its closest training point approaches zero.^17^, ^18^ The most common way to increase training density is to increase the number of training points used to sample the system but this is not necessarily desirable, as larger models require more data to train on, data which may be expensive to obtain. Also, larger training sets result in a larger correlation matrix **R**, such that more time is required to invert the correlation matrix when training the model, and the correlation matrix is more likely to have a high condition number.^40^ Fortunately, *training density can be increased for the water decamer described here, at no additional cost, by exploiting the physical indistinguishability of the water molecules within the cluster to redefine the sets of features in the training set*.

**Figure 2:**
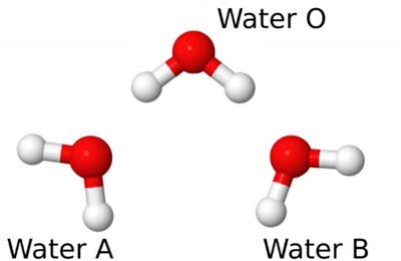
Example of a three‐molecule water system where the “central” molecule, *Water O*, is a hydrogen bond donor to the other two molecules, *Water A* and *Water B*. [Color figure can be viewed in the online issue, which is available at wileyonlinelibrary.com.]

An example of this concept is as follows. Consider a system of three water molecules, where one water molecule, *Water O*, is a hydrogen bond donor to the other two (*Water A* and *Water B*, as depicted in Fig. 2). A kriging model is centered on the oxygen of *Water O*, through the coordinate scheme outlined in Standard kriging models section, and trained to predict some property of the system based on the precise coordinates of the molecules *Water A* and *Water B*. The system is sampled via intermittent snapshots from a larger MD simulation but only snapshots that involve the molecule *Water O* acting as a hydrogen bond donor to two other water molecules are retained for this example system. This leads to a set of configurations that are similar to the molecular configuration displayed in Figure 2. Assume that *Water A* is defined to be the first molecule, as listed by the MD simulation's account keeping scheme and compared to *Water B*. Then the *Water A* molecule is equally likely to appear on the left‐hand side of Figure 2, as it is to appear on the right‐hand side. This is because the molecules in liquid water are free to move around the simulation box, and are therefore equally likely to exist in a given position with respect to the central molecule. Thus, a kriging model that requires the positions of *Water A* and *Water B* as inputs will have approximately *one half* of its training points representing the configuration where *Water A* is on the left and *Water B* is on the right of Figure 2, and approximately *one half* of its training points representing the opposite configuration. An alternative way of defining *Water A* and *Water B* would be to define the molecule on the left in Figure 2 as *Water A*, and the molecule on the right as *Water B*. Thus, under the new scheme, a kriging model that requires the positions of *Water A* and *Water B* as inputs will have *all* of its training points representing the configuration where *Water A* is on the left, and *Water B* is on the right, doubling the density of the sampling at no extra computational cost.

One such intuitive way to accomplish this idea for a system as complex as a water decamer is to define the features describing the system by distance to the water molecule at the center of the cluster. As the oxygen–oxygen radial distribution of liquid water has a multimodal oxygen probability density,^35^ defining the training set features by oxygen–oxygen distance will incorporate some of liquid water's local structure into the model, and result in features with less conformational freedom. In other words, by defining the features that represent the coordinates of the nine, noncentral water molecules by atomic index, as per the *Standard* definition scheme, nine sets of features that each span the entire radius of the cluster are obtained. Alternatively, by defining the features that represent the coordinates of the respective water molecules to be based on their molecular distance to the central water molecule, nine sets of features with different mean radii and ranges are obtained. Such a result reduces the conformational freedom within the description, and incorporates some of liquid water's local structure into the model (e.g., only sets of features within a certain mean radius are likely to represent water molecules of the first solvation shell), thereby increasing training point density at no additional computational cost. Defining features by radial distribution in this manner has been shown to significantly improve machine learning prediction errors in crystal structures,^41^ and to be comparable to the Coulomb matrix representation for large datasets of organic molecules.^21^

**Figure 3:**
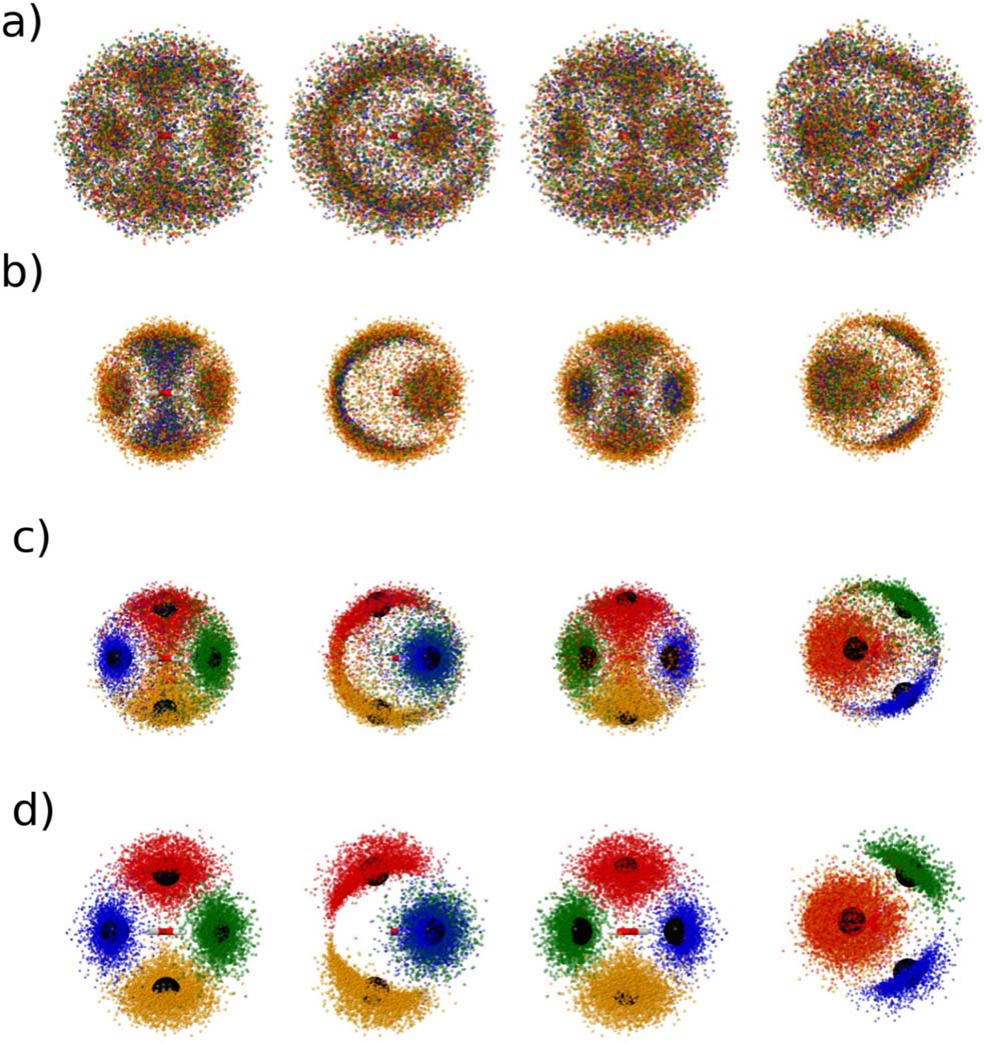
Spatial distribution of the first‐four, noncentral oxygens in the a) Standard, b) Distance, c) Structured 1a, and d) Structured 2a training sets. Each row of pictures represents the front, side, rear, and top view of the same distribution, respectively. Large black spheres are centered on the position of nodes. Colors blue, green, red, and orange represent the first, second, third, and fourth noncentral oxygens, as defined in the training set, respectively. In the *Standard* training sets, the features corresponding to these oxygens are defined by atomic index; in the *Distance* training sets, the features corresponding to the displayed oxygens are defined by distance from the central oxygen; in the *Structured* training sets, the features corresponding to the oxygens are defined by their respective node‐based structure scheme described in the text. [Color figure can be viewed in the online issue, which is available at wileyonlinelibrary.com.]

*Distance‐Defined (*from here on referred to as *Distance)* kriging models were created using the same oxygen‐ centered coordinate system as used for the *Standard* training set but the sets of features corresponding to each water molecule were defined by the OO distance between them and the oxygen of the central molecule. Figure 3a shows the spatial distribution of the first‐four, noncentral oxygens as defined by the *Standard* training set, and Figure 3b shows the spatial distribution of the first‐four, noncentral oxygens as defined by the *Distance* training set. Thus, the blue dots in Figure 3a display the spatial distribution of the decamer oxygens that have the lowest atomic index (as obtained from the MD simulation from which the clusters were sampled). Conversely, the blue dots in Figure 3b display the spatial distribution of the decamer oxygens that are nearest to the oxygen of the central water molecule. Similarly, the green, red, and orange dots in Figures 3a and 3b display the spatial distribution of the second, third, and forth oxygens of each feature definition scheme, respectively. Thus, as 5000 clusters were sampled, this means Figures 3a and 3b each display the spatial distribution of a total of 20,000 oxygens.

**Figure 4:**
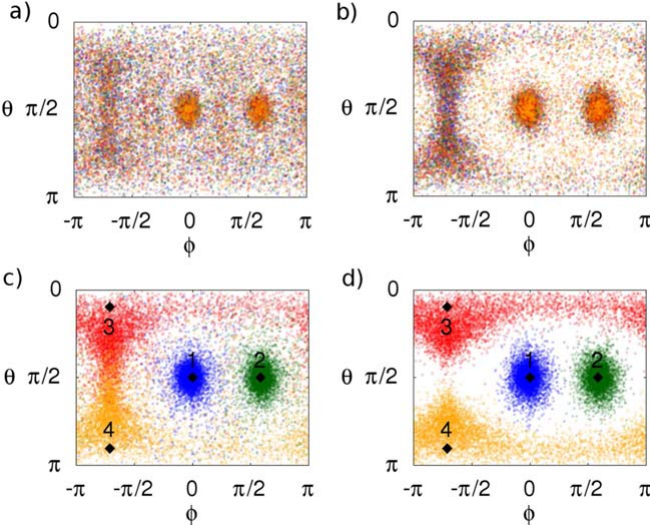
Projection of θϕ‐space for the first‐four noncentral oxygens in the a) Standard, b) Distance, c) Structured 1a, and d) Structured 2a training sets. Numbered diamonds represent position of nodes. Colors blue, green, red, and orange represent the first, second, third, and fourth noncentral oxygens, as defined in the training set, respectively. In the Standard training sets, the features corresponding to these oxygens are defined by atomic index; in the Distance training sets, the features corresponding to the displayed oxygens are defined by distance from the central oxygen; in the Structured training sets, the features corresponding to the oxygens are defined by their respective node‐based structure scheme described in the text. [Color figure can be viewed in the online issue, which is available at wileyonlinelibrary.com.]

It is seen that defining the training set features by distance (instead of atomic index) produces a much narrower spatial distribution of the first‐four, noncentral oxygen atoms, with a greater density of oxygen atoms around areas associated with the central molecule's hydrogen bonding. In fact, while there is only one combination of molecules that will result in the first four features being defined as the first solvation shell (i.e., the first four features are defined by the four atoms closest to the central oxygen), there are C4=9!4!5!=1269 possible combinations of molecules that the first four features might be defined by in the *Standard* method of defining the training set (e.g., first, third, eighth, and ninth closest, second, third, fourth, and seventh closest etc.). Thus, the *Distance* training set is expected to sample at a density 126 times higher than the *Standard* training set overall, when considering the features of the first solvation shell as equivalent (based on O—O distance). For clarity, Figures 4a and 4b display the overlapping projections of the first‐four, noncentral oxygens (as defined in the *Standard* and *Distance* training sets, respectively) onto a 2‐dimensional θϕ−space. Kriging results for the *Distance* model are presented the Results section.

### Structured kriging models

While *Distance* kriging models are intuitive, they still possess a large degree of conformational freedom. For condensed phase water, which is well known to possess local structure,^35^ improvements in prediction accuracy are possible by considering such structure in the design of the kriging models themselves. Hawe and Popelier reported significant prediction improvements by dividing the conformational space of a water dimer into seven overlapping regions, each endowed with an individual kriging model.^30^ Although effective for small systems, such a division of conformational space presents difficulties in higher dimension systems.

In both Figures 3b and 4b, increased oxygen density occurs near the central molecule's hydrogen atoms, as well as along a ridge at the rear of the central molecule, associated with the central oxygen's lone pairs. Thus, just as the *Distance* training set used the radial distribution of liquid water to guide the definition of features in the training set, it is possible to use the spatial distribution of liquid water in a similar manner. To do this, four nodes were placed on the vertices of a tetrahedron centered on the oxygen of the central water molecule, at a distance from the center that approximately corresponds to the first peak in the water radial distribution function. The coordinates of the nodes are given in Table 3, and the nodes are displayed in Figures 3c and 4c. Then, of the nearest four molecules to the center, that with the closest oxygen to the Node 1 was assigned as the first noncenter molecule to be listed in the training set. Of the remaining three molecules nearest to the center water molecule, that with the closest oxygen to Node 2 was assigned to be the next described in the training set and so on. The remaining five water molecules of the cluster were left defined by distance as per the *Distance* training sets. The resultant training set is named *Structured 1a*. Figure 3c displays the spatial distribution of the first four noncenter oxygens, as listed in the *Structured 1a* training set, and Figure 4c displays the overlapping projections onto the θϕ‐space of said oxygens.

**Table 3:** Position of nodes in structured training sets.

Node	*r* (Å)	Theta (degrees)	Phi (degrees)
1	2.65	90	0
2	2.65	90	105
3	2.65	17	−128
4	2.65	163	−128
5	3.18	90	172
6	3.18	90	−68
7	3.18	30	53
8	3.18	150	53

**Figure 5:**
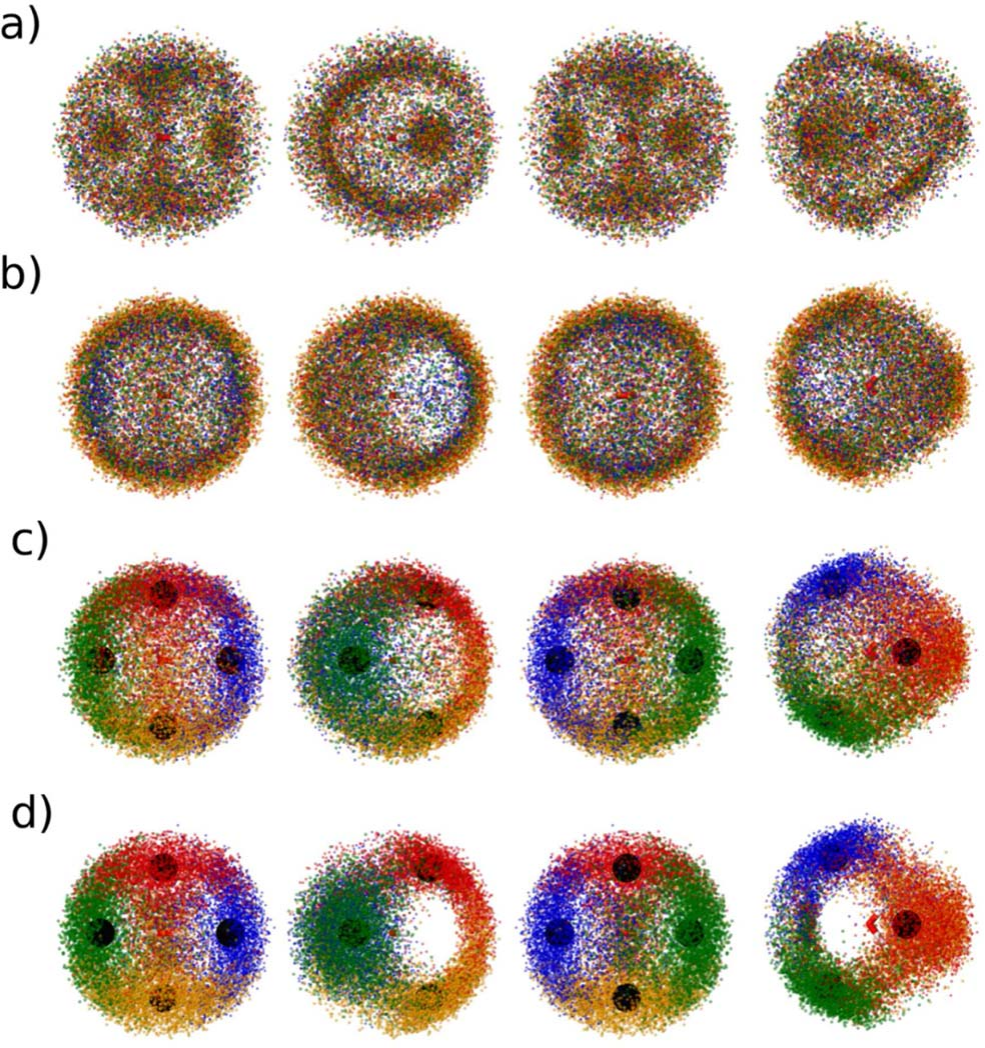
Spatial distribution of the fifth to eighth noncentral oxygens as listed in the a) Standard, b) Distance, c) Structured 1b, and d) Structured 2b training sets. Each row of pictures represents the front, side, rear, and top view of the same distribution, respectively. Large black spheres are centered on position of nodes. Colors blue, green, red, and orange represent the fifth, sixth, seventh, and eighth noncentral oxygens, as defined in the training set, respectively. In the *Standard* training sets, the features corresponding to these oxygens are defined by atomic index; in the *Distance* training sets, the features corresponding to the displayed oxygens are defined by distance from the central oxygen; in the *Structured* training sets, the features corresponding to the oxygens are defined by their respective node‐based structure scheme described in the text. [Color figure can be viewed in the online issue, which is available at wileyonlinelibrary.com.]

Nodes 1–4 were placed on the vertices of a tetrahedron centered on the oxygen of the central water molecule, at a distance from the center that approximately corresponds to the first peak in the water radial distribution function (see Fig. 3c to see how nodes 1–4 are positioned relative to the nearest four oxygens to the oxygen of the central water molecule). Nodes 5–8 were positioned similarly, at a slightly greater distance, as the structure they are positioned to account for occurs at a slightly greater distance (see Fig. 5c to see how nodes 5–8 are positioned relative to the fifth to eighth nearest oxygens to the oxygen of the central water molecule). Nodes 1–4 were used in the creation of *Structured 1a* and *Structured 2a* training sets (and their hydrogen‐ centered equivalents), whereas all eight nodes were used in the construction of *Structured 1b* and *Structured 2b* training sets (and their hydrogen‐ centered equivalents).

*Structured 2a* training sets were created as per the *Structured 1a* training sets, after removing the “nearest four molecules” restriction placed on the node allocation. Thus, the *Structured 2a* training set results in a larger variance of distance features $\left(R_{N}\right)$, but lower variance of angular features $\left(\theta_{N},\phi_{N}\right)$. Features corresponding to the remaining five oxygens were defined by distance to the center. Figures 3d and 4d display spatial distributions and θϕ‐space projections for the first four noncentral oxygens for the *Structured 2a* training set, respectively. Note that this means that figures representing the *Distance* and *Structured 1a* training sets contain the exact same points, defined differently. In contrast, figures representing the *Structured 2a* training set may contain different points (i.e., oxygens that were closer to nodes than any of the nearest four oxygens to the center were).

**Figure 6:**
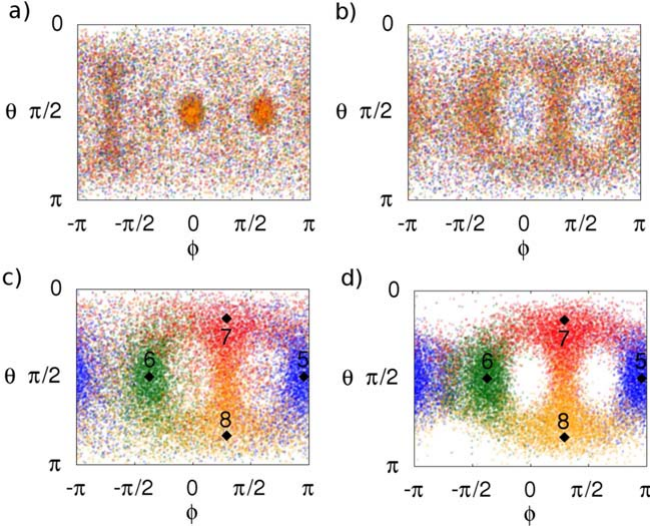
Projection of θϕ‐space for the fifth to eighth noncentral oxygens in the a) Standard, b) Distance, c) Structured 1b, and d) Structured 2b training sets. Numbered diamonds represent position of nodes. Colors blue, green, red, and orange represent the fifth, sixth, seventh, and eighth noncentral oxygens, as defined in the training set, respectively. In the Standard training sets, the features corresponding to these oxygens are defined by atomic index; in the Distance training sets, the features corresponding to the displayed oxygens are defined by distance from the central oxygen; in the Structured training sets, the features corresponding to the oxygens are defined by their respective node‐based structure scheme described in the text. [Color figure can be viewed in the online issue, which is available at wileyonlinelibrary.com.]

Beyond the first hydration shell, the spatial density of the surrounding water molecules is markedly different. Although regions of high density are not as well defined when compared to the spatial density of the nearest four molecules, they are still visible and tetrahedrally distributed about the central molecule (Fig. 5b). To account for this structure, four more nodes were added at the positions listed in rows 5–8 of Table 3. The new nodes were positioned at a distance close to the peak in the radial distribution of the next water molecule nearest to the center (i.e., the fifth closest water molecule to the center). In other words, where the first four nodes were positioned to account for the four water molecules closest to the central molecule, the next set of nodes were positioned at a distance that approximately corresponds to the expected distance of the fifth closest water molecule to the center. This choice was made because the decamer system is not large enough to incorporate the entire second solvation shell of a central water molecule. Using these nodes, a *Structured 1b* training set was constructed by applying the same feature defining method as used in *Structured 1a*, to the next four closest‐to‐center molecules in *Structured 1a*. Similarly, a *Structured 2b* training set was constructed by applying the same feature defining method as used in *Structured 2a*, but by extending the method to the full set of eight nodes. In both the *Structured 1b* and *Structured 2b* training sets, the remaining molecule was left as the last molecule in the training set. Figures 5c and 5d display the spatial distribution of the fifth through to eighth noncentral oxygens as listed in the *Structured 1b* and *Structured 2b* training sets, respectively, colored by node allocation, as well as the spatial distribution of the node positions. Similarly, Figures 6c and 6d display θϕ−space projections of the fifth through to eighth noncentral oxygens as listed in the *Structured 1b* and *Structured 2b* training sets, respectively, as well as the θϕ‐space projections of the fifth to eighth nodes.

### Hydrogen‐centered coordinate frame

In previous work, where kriging was applied to the prediction of atomic properties of amino acids, individual kriging models were created for each property of each atom, using an atom‐centered coordinate frame centered on the atom of interest, similar to the procedure outlined in the standard kriging models section above.^23^, ^24^, ^26^ When kriging has been used for the prediction of properties of water, however, each water molecule has been described as a whole, through a molecular coordinate frame.^27^, ^28^, ^30^ To investigate whether using an oxygen‐ centered coordinate frame is as effective as a hydrogen‐ centered coordinate frame when kriging atomic properties, each of the training sets listed above were also created using a coordinate frame centered on the *x*‐axis hydrogen (see Fig. 1). These training sets used the same feature definitions as the training sets that they were based on, but were centered on the *x‐*axis hydrogen, and defined their *x*‐axis to pass through the position of the central oxygen atom. Hydrogen‐ centered training sets were named as per the model they were based on, but with an added *H* (e.g., *Distance H*). A summary of all 12 feature definition methods is displayed in Table 4. Note, however, as the results obtained from the *Standard H* model were equivalent to the *Standard* model, only the results obtained from the *Standard* model will be displayed throughout the rest of this work.

**Table 4:** Overview of design differences between the 12 investigated models.

Model	Structuring method	Coordinate frame Center	Node allocation method
Standard	Defined by atom index	Oxygen	N/A
Standard H	Defined by atom index	Hydrogen	N/A
Distance	Defined by distance to central oxygen	Oxygen	N/A
Distance H	Defined by distance to central oxygen	Hydrogen	N/A
Structured 1a	4 nodes used. Then by distance to central oxygen	Oxygen	Restricted (first‐four)
Structured 1b	8 nodes used. Then by distance to central oxygen	Oxygen	Restricted (first‐four)
Structured 1aH	4 nodes used. Then by distance to central oxygen	Hydrogen	Restricted (first‐four)
Structured 1bH	8 nodes used. Then by distance to central oxygen	Hydrogen	Restricted (first‐four)
Structured 2a	4 nodes used. Then by distance to central oxygen	Oxygen	No restriction
Structured 2b	8 nodes used. Then by distance to central oxygen	Oxygen	No restriction
Structured 2aH	4 nodes used. Then by distance to central oxygen	Hydrogen	No restriction
Structured 2bH	8 nodes used. Then by distance to central oxygen	Hydrogen	No restriction

## Results

The performance statistics of the IQA kriging models are displayed in Table 5. As per the Standard Results section, each kriging model was trained and tested using fivefold cross validation with a 1:4 partitioning of the training set to the test set.

**Table 5:** Prediction statistics for the $E_{Self}$ and $E_{Inter}$ of the atoms of the central water molecule of a water decamer.

Property	Model	Mean Value	Range	MAE	*q* ^2^
Oxygen $E_{Self}$	Standard*	−196708.8	243.9	35.2 ± 1.3	0.00 ± 0.00
	Distance			11.4 ± 0.2	0.87 ± 0.01
	Distance H			21.7 ± 0.3	0.56 ± 0.02
	Structured 1a			11.2 ± 0.3	0.89 ± 0.00
	Structured 1b			11.0 ± 0.2	0.89 ± 0.00
	Structured 1aH			15.5 ± 0.3	0.77 ± 0.01
	Structured 1bH			15.8 ± 0.2	0.77 ± 0.01
	**Structured 2a**			10.6 ± 0.2	0.90 ± 0.00
	Structured 2b			11.7 ± 0.2	0.86 ± 0.01
	Structured 2aH			15.9 ± 0.3	0.77 ± 0.01
	Structured 2bH			19.1 ± 0.4	0.66 ± 0.02
Oxygen $E_{Inter}$	Standard*	−1540.6	407.7	50.2 ± 2.1	0.00 ± 0.00
	Distance			16.9 ± 0.5	0.87 ± 0.01
	Distance H			34.5 ± 0.7	0.48 ± 0.01
	Structured 1a			16.2 ± 0.3	0.88 ± 0.00
	Structured 1b			16.6 ± 0.4	0.88 ± 0.01
	Structured 1aH			24.7 ± 0.3	0.73 ± 0.01
	Structured 1bH			25.1 ± 0.8	0.72 ± 0.02
	**Structured 2a**			15.3 ± 0.6	0.91 ± 0.01
	Structured 2b			16.4 ± 0.2	0.87 ± 0.01
	Structured 2aH			24.6 ± 1.0	0.73 ± 0.03
	Structured 2bH			29.3 ± 0.5	0.61 ± 0.02
Hydrogen $E_{Self}$	Standard*	−736.1	121.6	18.1 ± 0.2	0.00 ± 0.00
	Distance			13.1 ± 0.4	0.41 ± 0.03
	Distance H			12.0 ± 0.2	0.55 ± 0.01
	Structured 1a			11.5 ± 0.3	0.56 ± 0.02
	Structured 1b			11.8 ± 0.5	0.52 ± 0.03
	Structured 1aH			10.5 ± 0.1	0.65 ± 0.01
	Structured 1bH			10.5 ± 0.2	0.65 ± 0.01
	Structured 2a			11.4 ± 0.2	0.59 ± 0.01
	Structured 2b			11.3 ± 0.3	0.57 ± 0.03
	**Structured 2aH**			10.3 ± 0.1	0.66 ± 0.01
	Structured 2bH			11.1 ± 0.1	0.61 ± 0.02
Hydrogen $E_{Inter}$	Standard*	−525.3	120.8	18.4 ± 0.5	0.00 ± 0.01
	Distance			13.6 ± 0.5	0.44 ± 0.04
	Distance H			8.1 ± 0.3	0.79 ± 0.01
	Structured 1a			11.6 ± 0.3	0.55 ± 0.02
	Structured 1b			11.8 ± 0.6	0.50 ± 0.04
	Structured 1aH			6.9 ± 0.2	0.84 ± 0.01
	Structured 1bH			6.8 ± 0.1	0.84 ± 0.00
	Structured 2a			11.5 ± 0.2	0.57 ± 0.01
	Structured 2b			11.6 ± 0.4	0.54 ± 0.03
	**Structured 2aH**			6.7 ± 0.0	0.85 ± 0.00
	Structured 2bH			7.0 ± 0.1	0.84 ± 0.01

The worst performing model for each property is indicated by *, while the best performing model is written **bold**. All energies are in kJ/mol, and uncertainties represent ± 1 standard deviation. Averages obtained from five 1000‐training‐point models, tested on 4000 test points each.

For all properties considered, kriging models that incorporated local structure outperformed the Standard models. The best performing model for the oxygen $E_{Self}$, oxygen $E_{Inter}$, hydrogen $E_{Self}$, and hydrogen $E_{Inter}$, returned MAE values 70%, 70%, 43%, and 64% lower than the *Standard* model of each property, respectively. In fact, even the worst performing models that incorporate local structure returned MAE values 38%, 31%, 28%, and 26% lower than the *Standard* model of the oxygen $E_{Self}$, oxygen $E_{Inter}$, hydrogen $E_{Self}$, and hydrogen $E_{Inter}$, respectively. Furthermore, Table 5 shows the importance of centring the coordinate frame on the atom of interest, with MAE values obtained from such training sets being on average 31% lower than errors obtained from training sets created with a coordinate frame centered on an atom other than the one the properties of interest come from. Finally, all *Structured* models, from a particular coordinate frame, for a particular property, outperformed their equivalent *Distance* model. On average, MAE values obtained from *Structured* models were 22% lower than errors obtained from the equivalent *Distance* models. Between *Structured* models, differences were less significant, with all oxygen‐ centered MAE's within ±2 standard deviations of each other. Similar was true of the *Structured 1aH*, *Structured 1bH*, and *Structured 2aH* hydrogen‐ centered models but the *Structured 2bH* models performed noticeably worse (although still better than the *Distance* models).

**Figure 7:**
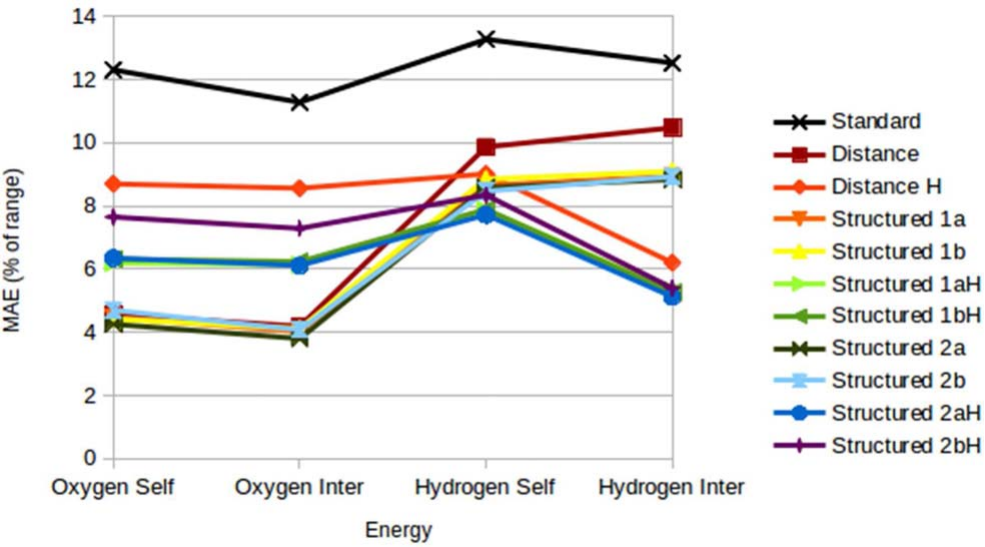
Mean absolute error of IQA energies as a percentage of range. [Color figure can be viewed in the online issue, which is available at wileyonlinelibrary.com.]

Figure 7 displays the MAE of energy predictions for each of the models, displayed as a percentage of the true range. Here, it can be seen that the best oxygen models predict with mean errors within 5% of the total range, and the best hydrogen models predict within 8%. In particular, the hydrogen $E_{Self}$ is predicted poorly relative to the hydrogen $E_{Inter}$, although the best performing hydrogen $E_{Self}$ model (*Structured 2aH)* still obtains a MAE 43% better than the worst performing hydrogen $E_{Self}$ model (*Standard),* and approximately 5.5% better when the difference is measured as a percentage of the range in the property.

**Figure 8:**
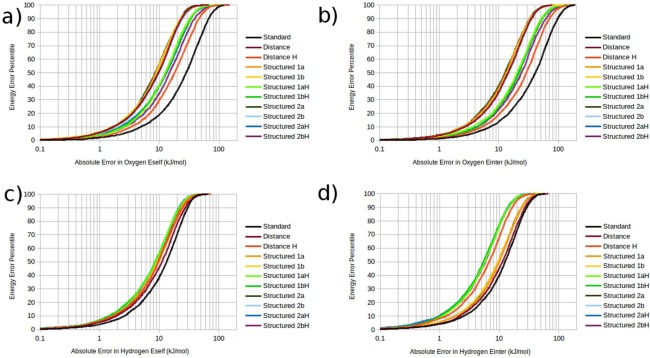
Error distributions of IQA kriging models. a) oxygen $E_{Self}$, b) oxygen $E_{Inter}$, c) hydrogen $E_{Self}$, d) hydrogen $E_{Inter}$. [Color figure can be viewed in the online issue, which is available at wileyonlinelibrary.com.]

Figure 8 displays the full distribution of energy property errors in S‐curve format. The *y*‐axis indicates what percentage of the test data corresponds to which prediction error given by the *x*‐axis. Similar to an error distribution histogram, the S‐curve format of error depiction is an intuitive means of quickly determining the characteristics of a distribution of errors. However, whereas a distribution histogram generally draws the eye to the mode (i.e., the value corresponding to the maximum of the distribution), the S‐curve presents the data in a way that makes ascertaining the various percentile values [such as the median value (50‐percentile), 90‐percentile value, 95‐percentile value etc.] convenient. From Figure 8 it can be seen that the best performing models for each oxygen based property possesses median and 95‐percentile MAE's that are approximately one third of the MAE of the worst performing model for that property, while the best performing models for each hydrogen based property possesses median and 95‐percentile MAE's that are approximately half to two‐thirds of the MAE of the worst performing model for that property.

**Figure 9:**
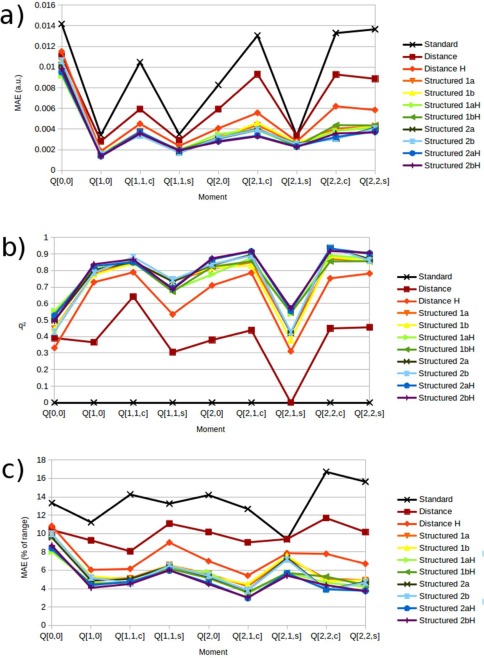
Validation results of hydrogen multipole moment kriging models showing a) mean absolute error, b) *q* ^2^ values, and c) mean absolute errors as a percentage of the range of predictions. [Color figure can be viewed in the online issue, which is available at wileyonlinelibrary.com.]

In addition to the IQA energies, kriging models for the QTAIM obtained electrostatic multipole moments were also obtained, for the charge (*Q*_00_), components of the dipole moment (*Q*_10_, *Q*_11c_, *Q*_11s_) and components of the quadrupole moment (*Q*_20_, *Q*_21c_, *Q*_21s_, *Q*_22c_, *Q*_22s_). Figure 9 displays the MAE, *q* ^2^, and MAE as a percentage of the range for the hydrogen kriging models. Again, the *Standard* model was uniformly the worst performing model, and the hydrogen‐centered models generally out‐performed their oxygen‐centered equivalents. Unlike the IQA results, the *Distance* models performed worse than the *Structured* models in general, regardless of the atom the coordinate frame was centered upon. The worst performing model in terms of MAE was the charge, with only the hydrogen‐centered *Structured* models returning an error less than 0.01a.u. Still, the best performing model for the charge, *Structured 1aH*, returned a MAE 35% lower than that of the *Standard* model. By using *Structured* kriging models, the *Q*_22c_ quadrupole component gained the most in accuracy, reducing errors by 75% when compared to the predictions of the *Standard* model.

Figure 9 also shows that the difference in prediction statistics between the various *Structured* models is much less than the difference between the *Structured* and *Distance* or *Standard* models. This implies that the specific means of accounting for the spatial distribution of the atoms surrounding the center is less important than the fact it is accounted for in the first place. *Consideration of the specific spatial structure of the system beyond the first solvation shell demonstrated no further improvement in prediction accuracy*. Note that the difficulty of a kriging problem is a function of the range the model has to predict over, and as such a property with a slightly larger MAE but a significantly larger range may appear much better when judged using *q* ^2^ or the MAE as a percentage of the range (Figs. 9b and 9c, respectively). This is particularly evident with the *Q*_11c_ component of the dipole moment (corresponding to the approximate O—H···O axis), which has a MAE approximately twice that of the *Q*_10_ and *Q*_11s_ moments in Figure 9a, but superior *q* ^2^ values. Thus, when comparing the prediction results of different models, from different coordinate frames, it is important to compare across the complete set of multipole moments (i.e., consider all three dipole components, or all five quadrupole components, together), and across the set of three validation metrics.

**Figure 10:**
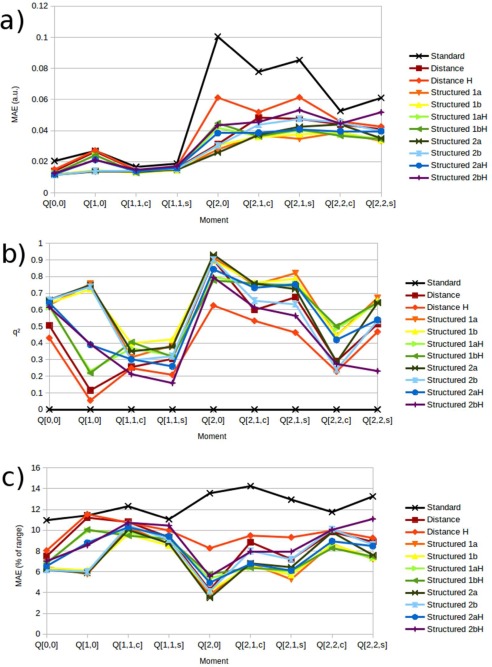
Validation results of oxygen multipole moment kriging models showing a) mean absolute error, b) *q* ^2^ values, and c) mean absolute errors as a percentage of the range of predictions. [Color figure can be viewed in the online issue, which is available at wileyonlinelibrary.com.]

Figure 10 displays the MAE, *q* ^2^, and MAE as a percentage of the range for the oxygen's charge, dipole, and quadrupole kriging models. Again, the *Standard* kriging model is consistently the worst, although here the oxygen‐ centered *Distance* model outperforms several of the hydrogen‐centered models for a range of moment components. Unlike for the hydrogen, the charge of the central oxygen was the best predicted moment in terms of the MAE but it is also seen that there are other multipole moments that outperform it in terms of *q* ^2^ and MAE as a percentage of range. Thus, the larger MAE's seen in the higher rank moments, and in particular for the quadrupole moment, are a consequence of their larger property ranges and not some fault of the models. Such results highlight the importance of considering multiple metrics when validating machine learning models. Again, the *Structured 1a* and *Structured 1b* models produce similar results, as do the Structured *2a* and *Structured 2b* models, however, unlike the hydrogen results, the oxygen multipole moments seem to respond significantly better to a local (i.e., oxygen‐ centered) coordinate frame. In fact, for the *Q*_22s_ component, the *Structured 2bH* model is outperformed by both *Distance* models. This is possibly due to the extensive range each *R_N_* feature may possess under this feature definition scheme, particularly when centered on the hydrogen atom, which is off center in the cluster.

Although machine learning algorithms have been used to predict the multipole moments of water previously^21^, ^27^, ^28^ a clear comparison between results is difficult due to different reporting techniques and system specifications. Handley et al.^28^ applied kriging, radial basis function neural networks, and multilayer perceptrons, as well as a variety of combinations of the three, to the prediction of the multipole moments of a water at the center of small water clusters, using a molecular local frame. For the water pentamer, the best dipole moment predictions were obtained through kriging, with a MAE of 0.066 a.u. This is significantly larger (about 5 times) than the MAE across all dipole moment components of 0.0137 a.u. for oxygen and 0.00214 a.u. for hydrogen obtained here, despite the decamer being a larger system. In a separate work, Handley and Popelier^27^ obtained a mean error in the charge of the central water of a hexamer cluster of 0.0091 a.u., which is similar to the MAE error obtained for the best performing decamer model presented here. In addition, an average absolute dipole moment error of 0.077 a.u. was reported (over six times higher than the best performing models here). For their validation set, no component of the dipole moment obtained a correlation coefficient above 0.25 (compared to over 0.9 for the best performing models here). Moreover, Bereau et al.^21^ investigated the application of machine learning models trained on a variety of small organic molecules to chemical problems, including the prediction of multipole moments. As the water monomer is substantially different in its properties when compared to the other small molecules considered, predictions on the water monomer were poor, with an absolute error of 0.26 a.u. on the charge, a MAE of 0.14 a.u. across the components of the dipole moment, and a MAE of 0.356 a.u. across the components of the quadrupole moment (all one order of magnitude higher than the results obtained here). As the objective of Bereau et al.'s work was to create a machine learning model that can predict multipole moments accurately across a very wide range of different molecules, as opposed to creating a model specifically for water clusters, a direct comparison is not fair. However, it is interesting to note the difference in results that the two different approaches give.

## Conclusion

Various methods of training set construction of the water decamer were considered, with an intention to better incorporate known local structure into kriging models. By exploiting the physical indistinguishability of the water molecules within the cluster to redefine the features of the training set, we show that training density can be increased, at no additional cost. Although all training sets contained the same conformational information, careful definition of training set features to account for local structure led to improvements in mean absolute prediction error of up to ∼75% for certain properties. In addition, it was found that, for the properties investigated, centring the coordinate frame of a kriging model on the atom‐of‐interest also leads to improved prediction accuracy. Although no single method of accounting for local molecular structure performed best for every property considered, the results presented here suggest that, when using machine learning to model and predict chemical properties, careful consideration of the spatial distribution of the system around the atom of interest is an essential requirement for the reduction of prediction errors, particularly in system which possess large amounts of conformational freedom. Thus, at the very least, when considering the atoms at the center of a cluster, one should attempt to design training sets to account for the structure of the first solvation shell of the atom or molecule‐of‐interest, and center kriging models on the atom‐of‐interest where possible. Such a procedure requires no extra *ab initio* data, however was shown to reduce prediction errors by up to ∼75% for certain properties. Finally, it was shown that the kriging method employed here appears to perform very well when compared to a short review of similar, smaller systems—although different model parameterization and model validation methods make a true comparison not feasible.
